# Factors associated with low contraceptive use amongst vulnerable mothers in South West State, Nigeria

**DOI:** 10.4102/phcfm.v12i1.2552

**Published:** 2020-09-18

**Authors:** Obasanjo A. Bolarinwa, Olalekan S. Olagunju, Akintayo T. Olaniyan

**Affiliations:** 1 Department of Public Health Medicine, School of Nursing and Public Health, College of Health Sciences, University of KwaZulu-Natal, Durban, South Africa; 2 Department of Demography and Social Statistics, Faculty of Social Sciences, Obafemi Awolowo University, Ile-Ife, Nigeria

**Keywords:** family type, living arrangement, family planning, vulnerable, south west, Nigeria

## Abstract

**Background:**

Young mothers tend to be more prone to high maternal and perinatal risks and are thus deemed vulnerable to adverse sexual and reproductive health rights (SRHR) in terms of their right to choose contraceptives of their choice to enhance their maternal well-being and childbirth spacing should be well discussed. Achieving sufficient SRHR may be averted if the use of family planning by disadvantaged groups is not given required attention.

**Aim:**

This study aimed to identify and analyse the factors associated with the low use of contraceptives amongst vulnerable women in the South West region in Nigeria.

**Setting:**

The study area was purposively chosen to capture contraceptive use amongst vulnerable women in Osun State, Nigeria.

**Methods:**

A primary data collection was done in three senatorial districts of Osun State, Nigeria, with 140 respondents each to give a total of 420 respondents. Collected data were analysed using univariate, bivariate and multivariate measures.

**Results:**

The result showed a magnitude of association and relationship at both levels of analyses. Living arrangements and family types were 89% and 88.3%, respectively, associated with family planning use. In the same vein, living arrangement and family types were also statistically significant at *p <* 0.05with an odds ratio of 0.23 (95% CI: 0.1184–0.4583) and an odds ratio of 0.35 (95% CI: 0.1756–0.6970) with family planning use, respectively.

**Conclusion:**

We concluded that policies and interventions to accelerate and encourage contraceptives use amongst vulnerable mothers in South West, Nigeria should be targeted at those whose husbands lived elsewhere and those whose husbands have more than one wife.

## Introduction

An estimate of more than 225 million women in the developing world were unable to access and use family planning or contraception (FP/C); this is also the cause of high fertility levels in African countries^1^ where Nigeria is not excluded with a total fertility rate (TFR) of 5.3 per woman, and the annual population growth rate is 3.2% with an estimated population of 192 million.^2,3,4^ It is agreed that the increased spending on family planning has a compelling long-term interest in increasing human resources and family happiness.^5^ In order to reap the dividend from these investments and meet the third goal of the 2030 sustainable development goals (SDGs), the disadvantaged groups that are key to Nigeria’s population growth must be recognised.^6^

Young mothers seem to be more exposed to high maternal and perinatal risks and are seen as being vulnerable. Therefore, their sexual and reproductive health right (SRHR) in terms of their right to the choice of contraception should be well addressed to improve their maternal and child health outcomes. It was also noted that most births in sub-Saharan African countries take place in the union, and most of such deliveries are mistimed or unintended. This is more prominent in Eastern & Western Africa where the unmet need for family planning is the highest.^7,8,9,10,11^

Reduction in the non-use of contraceptives has a uniquely wide range of demographic, economic and environmental benefits, in addition to its well-documented health advantages for women and children, and this is because of its direct link to family sizes and population change.^12^ Previous studies have noted that increasing contraceptive use in countries with high fertility rates has the potential of averting about 32% of all maternal deaths and 10% of childhood deaths.^13^

Considering Nigeria’s maternal rate of 157 per 100 000 livebirths^13^ and child mortality rates of approximately 143 per 1000 children, surviving up to 12 months of age is particularly high compared with other regions that are economically developed or are on the path of economic development.^14^ This large variability of non-use of family planning is higher amongst young mothers in the south west region with an average unmet need for contraception at 28% compared with other regions regions with much lower unmet need for contraception,^3^ hence the young mothers could delay the progress of the country in harnessing or reaping its demographic dividend and adequate family planning use has full capacities of averting the high rate of maternal and child mortality.

Families can be classified on the basis of several dimensions; for example, on the basis of marriage type, families can be monogamous or polygamous which is also valid based on other classifications.^15^ It is estimated that about 124 million couples, mainly in third world countries, do not use effective contraceptives, despite their desire to limit or space the birth of their children,^8^ with 20% of Nigerian couples having unmet needs for contraceptives despite being sexually active.^13^ This has a lot to do with cultural and societal values the family accepts.^10^ In varying degrees, polygamy seems to be the norm in most African societies, with some 45% – 55% of women living in polygamous family settings.^10,16^

There is also evidence of a close relationship between health, well-being and living arrangements.^17^ Literature also argued the assertions that the decision-making on contraception comes within the domain of women but this rather depends on the type of union or how the living arrangement is structured.^18,19^

Despite significant global resources and investments that are being directed to Nigeria to meet the current demand for family planning services,^20^ the level of contraceptive use amongst married or in-union women is lower compared with other developing countries.^21^ The gaps in attaining adequate sexual and reproductive health may be delayed if family planning use is not encouraged amongst vulnerable groups.^22^ Hence, there are critical needs to examine the family type, living arrangement and contraceptive use amongst vulnerable mothers in South West, Nigeria.

## Aim

This study aimed to identify and analyse the factors associated with the low use of contraceptives amongst vulnerable women in the South West region in Nigeria.

## Methodology

### Study design

The study was quantitative research, which employed a cross-sectional descriptive survey design to elicit information on family planning use, family types and living arrangements amongst vulnerable mothers in Osun State, Nigeria using a structured questionnaire.

### Setting

Osun State is an inland state in south-western Nigeria. Its capital is Osogbo. It is bounded in the north by Kwara State. The state enjoys a tropical climate characterised by two seasons: rainy season (March to October) and dry season (November to March).

### Data collection

The study population comprised young mothers within the reproductive age of 15–30 years who already had a child in the last year. The study area was purposively chosen to capture contraceptives use and associated factors amongst this group. The data collection was done in three senatorial districts of Osun State, Nigeria. Yamane’s formula for estimating sample size was used to estimate sample size for this study,^23^ and the arrived estimated sample size was 420, including a 5% margin of error or non-response. 140 respondents were interviewed in each senatorial district using a structured questionnaire and a multi-stage sampling procedure to give 420 respondents altogether.

### Analysis

The analysis was performed with STATA 14 software. The data were analysed adopting univariate, bivariate and multivariate measures. Univariate analysis was used for the frequency distribution of selected socio-economic and demographic variables that are related to this study in the dataset displayed. A Chi-square test and logistic regression analysis were employed to show an association, relationship and predict the likelihood between the independent variables, which are family type and living arrangement on the dependent variable, which is family planning use.

### Ethical consideration

The researcher ensured that the informed consent of the respondents was sought by explaining the purpose of the study; anonymity was maintained by not including their names, and confidentiality was assured. Before administering the questionnaire, a copy of the research protocol was submitted for a full review to the Research and Ethics committee of Institute of Public Health, Obafemi Awolowo University, Ile-Ife (IPH, OAU) and approval for this study was obtained with IPHOAU/12/1446.

## Results

Table 1 represents the respondents’ and husbands’ socio-demographic characteristics. The majority of the respondents were between ages 25 and 29 years, with the mean age of 26 years. Most of the respondents have more than primary education. More than half of the respondents have religion as Christianity (66.4%). Almost all the respondents were employed (90.7%). The living arrangement shows that 80.5% of the respondents were living with their husbands, whilst 19.5% were living elsewhere. The majority of the respondent’s husband (81.2%) married only once, whilst 18.8% married more than once. Children ever born (CEB) shows that 60.7% of respondents had 1–2 children, whilst 39.3% had three or more children with the mean CEB of 2 children. The use of family planning shows that 81.7% of respondents were currently using any method of family planning.

**TABLE 1:** Respondents’ and husbands’ socio-demographic characteristics (*N* = 420).

Variables	Respondents†		Husbands‡	
	Freq	Percent	Freq	Percent
**Age**				
Below 20	19	4.5	-	-
20–24	99	23.6	-	-
25–29	252	60	-	-
30+	50	11.9	-	-
25–29	-	-	96	22.8
30–34	-	-	173	41.2
35–39	-	-	110	26.2
40+	-	-	41	9.8
**Level of education**				
No formal education	15	3.6	2	0.5
Primary	44	10.5	9	2.1
Secondary	177	42.1	138	32.9
Post-secondary	184	43.8	271	64.5
**Religion**				
Christianity	279	66.4	279	66.4
Islam	139	33.1	141	33.6
Traditional	2	0.5	-	-
**Employment status**				
Unemployed	39	9.3	30	7.1
Employed	381	90.7	390	92.9
**Living arrangement**				
Living with husband	338	80.5	-	-
Husband live elsewhere	82	19.5	-	-
**Family type**				
Monogamous	341	81.2	-	-
Polygamous	79	18.8	-	-
**Children ever born**				
*Mean CEB = 2*				
1–2	255	60.7	-	-
3+	165	39.3	-	-
**Currently using FP**				
No	77	18.3	-	-
Yes	343	81.7	-	-

† , Mean age = 26 yrs

‡ , Mean age = 33 yrs

CEB, children ever born; FP, family planning.

In contrast, the respondent’s husbands socio-demographic characteristics show that majority of them were between ages 30 and 34 years with the mean age of 33 years. The majority of the respondent’s husbands have more than primary education (secondary 32.9% and post-secondary 64.5%). More than half of the respondent’s husbands have their religion as Christianity (66.4%). Almost all the respondent’s husbands were employed (92.9%).

Table 2 presents the bivariate association and logistic relationship between living arrangement, family type and family planning used amongst women who already had a child in the last year. The result above showed a significant association between living arrangement and use of family planning in that 89% of women who were living with their husband and 51.2% of women whose husbands were living elsewhere were using family planning. With regard to family type, the table shows that 88.3% of women whose husbands had only one wife and 53.2% of women whose husbands had more than one wife were using family planning. The table also reveals that there is a significant association between the use of family planning, living arrangements and family type as *p* < 0.05.

**TABLE 2:** Bivariate association and logistic regression of living arrangement, family type and use of family planning (*N* = 420).

Variable	Family planning use								
	Bivariate						Logistic regression		
	No		Yes		Chi-square		Odd ratio	*p*-value	CI
	*n*	%	*n*	%	*χ*^2^	*p*-value			
**Living arrangement**									
Living with husband	37	11.0	301	89.0	63.09	0.000*	RC	0.000*	-
Husb. live elsewhere	40	48.8	42	51.2	-	-	0.23	-	0.1184–0.4583
**Family type**									
Monogamous	40	11.7	301	88.3	52.79	0.000*	RC	0.003*	-
Polygamous	37	46.8	42	53.2	-	-	0.35	-	0.1756–0.6970

RC, recode.

Logistics regression analysis was carried out to assess the net effect of living arrangements and family type on the use of family planning. Table 2 also shows that women whose husbands live elsewhere are 0.23 less likely to use family planning with (95% CI: 0.1184–0.4583); in the same vein, women whose husbands had more than one wife were also 0.35 less likely to use family planning with (95% CI: 0.1756–0.6970). Both living arrangements and family type were statistically significant in the study at *p* < 0.05.

## Discussion

This study also revealed that family type and the living arrangement were significantly associated with the use of family planning. The research shows that women whose husbands have more than one wife were less likely to use family planning compared with women whose husbands had only one wife. This may be due to marital conflict amongst wives with regard to the birth order or sex preference.^11^ Also, women whose husbands were living elsewhere were less likely to use any method of family planning. This may be because women were not exposed to frequent sexual intercourse compared with women who were living with their husbands.^10^

The need to subscribe to contraceptives use amongst vulnerable young mothers who lived away from their husbands and who are in the polygamous union might not be there because of their infrequent sexual activities as the husbands are often not available. This study unveiled this peculiar group of non-family planning users to help scale up the adoption in South West State, Nigeria.

## Conclusion

Although the prevalence level of contraceptive use amongst sexually active men and women is low in Nigeria,^8,10,20^ we studied variations by living arrangements and family types in the use of any family planning method amongst young mothers. The use of any family planning methods was high amongst young mothers living with their husbands and whose husbands have one wife. The family type was significantly associated with the use of family planning. Women whose husbands were living elsewhere were less likely to use a method of family planning. Also, the living arrangement was significantly associated with the use of family planning. Young mothers whose husbands have more than one wife were less likely to use family planning.^11^ Adoption of a family planning practice campaign that considers living arrangements and family types will further scale up contraceptive use amongst young women.^5,19^
